# Catalytic and structural properties of pheophytinase, the phytol esterase involved in chlorophyll breakdown

**DOI:** 10.1093/jxb/erx326

**Published:** 2017-09-23

**Authors:** Luzia Guyer, Kathrin Salinger, Undine Krügel, Stefan Hörtensteiner

**Affiliations:** Institute of Plant and Microbial Biology, University of Zurich, Zollikerstrasse, Zurich, Switzerland

**Keywords:** 3D model, catalytic triad, chlorophyll breakdown, phytol, senescence, serine esterase, site-directed mutagenesis, substrate specificity

## Abstract

During leaf senescence and fruit ripening, chlorophyll is degraded in a multistep pathway into linear tetrapyrroles called phyllobilins. A key feature of chlorophyll breakdown is the removal of the hydrophobic phytol chain that renders phyllobilins water soluble, an important prerequisite for their ultimate storage in the vacuole of senescent cells. Chlorophyllases had been considered for more than a century to catalyze dephytylation *in vivo*; however, this was recently refuted. Instead, pheophytinase was discovered as a genuine *in vivo* phytol hydrolase. While chlorophyllase acts rather unspecifically towards different porphyrin substrates, pheophytinase was shown to specifically dephytylate pheophytin, namely Mg-free chlorophyll. The aim of this work was to elucidate in detail the biochemical and structural properties of pheophytinase. By testing different porphyrin substrates with recombinant pheophytinase from *Arabidopsis thaliana* we show that pheophytinase has high specificity for the acid moiety of the ester bond, namely the porphyrin ring, while the nature of the alcohol, namely the phytol chain in pheophytin, is irrelevant. *In silico* modelling of the 3-dimensional structure of pheophytinase and subsequent analysis of site-directed pheophytinase mutant forms allowed the identification of the serine, histidine, and aspartic acid residues that compose the catalytic triad, a classical feature of serine-type hydrolases to which both pheophytinase and chlorophyllase belong. Based on substantial structural differences in the models of Arabidopsis pheophytinase and chlorophyllase 1, we discuss potential differences in the catalytic properties of these two phytol hydrolases.

## Teaser

Pheophytinase comprises a catalytic-triad of residues and shows limited but physiologically relevant substrate specificity towards magnesium-free chlorophyll derivatives due to its lid domain.

## Introduction

Chlorophyll breakdown is one of the most obvious signs of leaf senescence and fruit ripening. The resulting yellowing of leaves can be observed every autumn and the change in color of fruits indicates their stage of ripening. During these processes, chlorophyll is broken down in a multistep pathway. This pathway is now termed the PAO/phyllobilin pathway (Hörtensteiner and Kräutler, 2011; Christ and Hörtensteiner, 2014), acknowledging that the core enzymatic step of chlorophyll breakdown is catalyzed by pheophorbide *a* oxygenase (PAO) (Pružinská *et al.*, 2003), which ultimately determines the basic linear tetrapyrrolic structure of the end catabolites of breakdown called phyllobilins (Kräutler, 2014).

The breakdown of chlorophyll starts with chlorophyll *a* via the loss of the central Mg^2+^ ion, resulting in pheophytin *a*. Many attempts have been undertaken in the past to identify the underlying mechanism of Mg-dechelation (Suzuki and Shioi, 2002). Only very recently was it shown that stay-green proteins (SGRs), which have been known about in the molecular sense for a decade (Armstead *et al.*, 2006; Armstead *et al.*, 2007; Park *et al.*, 2007; Ren *et al.*, 2007) and whose absence in respective mutants cause stay-green phenotypes during leaf senescence and fruit ripening in many plant species (Barry, 2009; Hörtensteiner, 2009), catalyze Mg-dechelation *in vitro* and *in vivo* (Shimoda *et al.*, 2016). The second step of chlorophyll degradation is the dephytylation of pheophytin *a*, leading to the formation of pheophorbide *a*, the substrate of PAO. In several plant species this step is catalyzed by pheophytinase (PPH) and it has been shown that absence of PPH in leaves results in a stay-green phenotype (Morita *et al.*, 2009; Schelbert *et al.*, 2009; Guyer *et al.*, 2014; Zhang *et al.*, 2016). The phytol chain anchors pigments within the chlorophyll *a*/*b* binding complexes in thylakoid membranes and loss of the phytol chain from pheophytin *a* is considered a prerequisite for the release of pheophorbide *a* from the membrane for further degradation by downstream enzymes (Schelbert *et al.*, 2009). Oxygenolytic opening of the porphyrin ring by PAO leads to a transient intermediate, red chlorophyll catabolite, which is further reduced by red chlorophyll catabolite reductase to *primary* fluorescent chlorophyll catabolite (*p*FCC), the first colorless and non-photodynamic degradation product of chlorophyll. After subsequent site-specific hydroxylation by TIC55 (Hauenstein *et al.*, 2016), FCCs are exported from senescing chloroplasts and species-specifically modified at different peripheral side chain positions in the cytosol (Christ and Hörtensteiner, 2014). Finally they are imported into the vacuoles of senescing cells and stored as phyllobilins (Kräutler, 2014).

During the last decade the process of dephytylation during chlorophyll breakdown has received much attention. Since its first description more than a century ago (Willstätter and Stoll, 1913), chlorophyllases (CLHs) have been considered to catalyze phytol cleavage during chlorophyll breakdown. Chlorophyll *a* was assumed to be first dephytylated to chlorophyllide *a* before the central magnesium ion is lost (Matile *et al.*, 1999). The first molecular cloning of CLHs in 1999 (Jakob-Wilk *et al.*, 1999; Tsuchiya *et al.*, 1999) seemed to support the role of CLHs in chlorophyll breakdown (Takamiya *et al.*, 2000; Azoulay-Shemer *et al.*, 2011; Takamiya *et al.*, 2000); however in *Arabidopsis thaliana*, neither of the two CLHs localize to plastids (Schenk *et al.*, 2007; Hu *et al.*, 2015). Furthermore, double mutants deficient in both genes are not compromised in their ability to degrade chlorophyll during senescence (Schenk *et al.*, 2007). Instead, PPH has been identified as an enzyme that catalyzes pigment dephytylation *in vivo* (Schelbert *et al.*, 2009). PPH is a chloroplast-localized α/β hydrolase with a key serine residue in its active site. The absence of PPH in different plant species results in stay-green phenotypes (Morita *et al.*, 2009; Schelbert *et al.*, 2009; Ren *et al.*, 2010). Interestingly, PPH mutants accumulate pheophytin *a in vivo*, and *in vitro*, PPH was shown to specifically hydrolyze pheophytin and does not accept chlorophyll as a substrate (Schelbert *et al.*, 2009; Guyer *et al.*, 2014). This specificity is intriguing and has been discussed as a means to biochemically separate the pathway of chlorophyll breakdown from the pathway of chlorophyll biosynthesis (Schelbert *et al.*, 2009; Hörtensteiner, 2013). Indeed, chlorophyllide, the product of CLH activity, is also the penultimate intermediate of chlorophyll biosynthesis. Thus, preceding Mg-dechelation over dephytylation biochemically separates the anabolic pathway from the catabolic pathway. In addition, biotechnological mistargeting of Arabidopsis CLH1 to the chloroplast resulted in a severe light-dependent cell death phenotype, indicating that chlorophyllide formation from chlorophyll is highly phototoxic (Hu *et al.*, 2015). This might explain the requirement of PPH as the core phytol hydrolytic enzyme during chlorophyll breakdown. However, the underlying molecular mechanism determining the high substrate specificity of PPH towards pheophytin remains unknown.

This study aims to provide a detailed biochemical determination of the properties of Arabidopsis PPH. From its primary amino acid composition, PPH belongs to the α/β hydrolases, a broad family of enzymes including many esterases and lipases that are composed of eight parallel β sheets and that form a catalytic triad in their active site (Schrag and Cygler, 1997; Rauwerdink and Kazlauskas, 2015). It is also known that esterases show high substrate preference with either specificity towards the alcohol or the acid moiety of the ester bond (Fojan *et al.*, 2000). Here we show important biochemical traits of PPH and identified structural characteristics of porphyrins that define the substrate specificity of PPH. In addition, by using structural models of PPH that are based on known crystal structures of other hydrolases, we identify the amino acid residues forming the catalytic triad using site-directed mutagenesis.

## Material and methods

### Cloning and expression of PPH

A truncated *PPH* fragment (*ΔPPH*), lacking the predicted transit peptide of 46 amino acids encoded by the first 138 bases (Schelbert *et al.*, 2009), was amplified with Phusion polymerase (New England Biolabs) using the primers PPH_LIC_fw and PPH_LIC_rv (see Supplementary Table S1 at *JXB* online). The vector pMCSG29 (Eschenfeldt *et al.*, 2010) was digested with *Sma*I and after gel purification of both vector and PCR product, ligation-independent cloning (LIC) was performed as published (Eschenfeldt *et al.*, 2009; De Rybel *et al.*, 2011), with the following adaptations: the T4 polymerase reaction (New England Biolabs) was supplemented with 0.2 mg ml^-1^ BSA (New England Biolabs) and 5 mM dithiothreitol. dTTP was added to a concentration of 1 mM to the PCR reaction and dATP at 1 mM to the vector reaction. Reactions were incubated for 90 min at 22°C and stopped by incubation for 20 min at 75°C. Subsequently, T4-treated vector and PCR product were combined and incubated for 40 min at room temperature. NEB 10-beta competent *E. coli* (High Efficiency) cells (New England Biolabs) were transformed with the construct, named ΔPPH-MBP. After verifying sequence accuracy by sequencing, ΔPPH-MBP was introduced into *E. coli* BL21 (DE3). Point mutations were introduced by PCR using primers as listed in Supplementary Table S1. All reactions were carried out using PCR Extender Polymerase (5Prime). The S221A mutant version of PPH was produced with Phusion polymerase (New England Biolabs) from a plasmid expressing MBP-ΔPPH_S221A_ (Schelbert *et al.*, 2009) using the primers PPH_LIC_fw and PPH_LIC_rv (Supplementary Table S1). All resulting PCR products were treated with T4 polymerase as described above, LIC cloned into pMCSG29, and finally introduced into *E. coli* DH5α. Point mutations were verified by sequencing and subsequently the constructs were transferred into *E. coli* BL21 (DE3) (Thermo Fisher Scientific) for protein expression.

For recombinant protein expression, *E. coli* BL21 (DE3) harboring the different pMCSG29 constructs were grown at 37°C in Luria Broth (LB) medium supplemented with 2 g L^-1^ glucose. With an optical density at 600 nm of 0.5–0.6 protein expression was induced by adding 1 mM isopropyl β-D-1-thigalactopyranoside and cultures were grown overnight at 20°C. Cells were collected by centrifugation and resuspended in 20 mM Tris-HCl at pH 8 and 200 mM NaCl. Disruption of the bacterial cells was performed by using a French Press (Constant Cell Disruption System; Constant Systems) at 150 MPa. Soluble protein fractions were separated by centrifugation, supplemented with 10% glycerin [v/v] and kept at -80°C until further use. As a control, cells containing the empty vector pMCSG29 were used. Presence of expressed proteins was analyzed by SDS-PAGE on 4–15% Mini-PROTEAN TGX gels (BioRad) using Coomassie blue staining or by immunoblot analysis. For the latter, membranes were hybridized with either anti-MBP antibodies (New England Biolabs) or custom-made anti-PPH antibodies, which were raised in rabbits against a keyhole limet hemocyanin-conjugated Arabidopsis PPH peptide, 127-VGSFHYEKQLTDLGRD-142 (AgriSera).

### Activity assays

All activity assays were performed with substrates dissolved in acetone and at a final acetone concentration of 10% [v/v] in the reaction mixtures. Pheophytin *a* was produced from chlorophyll *a* (Livchem Logistics) as previously described (Schelbert *et al.*, 2009). The standard assay was performed in reaction buffer, comprising 0.1 M Bicine-KOH at pH 8.5 and 1 mM EDTA, at 34°C for 60 min. Reactions were stopped by adding two volumes of acetone, followed by centrifugation for 2 min. Reactions were analyzed by HPLC as published (Langmeier *et al.*, 1993).

Time dependency of PPH activity towards pheophytin *a* or chlorophyll *a* as substrates was performed under standard assay conditions with 375 µg ml^-1^ PPH and 100 µM substrates for up to 120 min. The optimal temperature of PPH was determined by incubating 10 µM pheophytin *a* with150 µg ml^-1^ PPH in reaction buffer at different temperatures. For determining the optimal pH, 150 µg ml^-1^ PPH was incubated at 34°C with 10 µM pheophytin *a* in buffers at different pH, namely 0.1 M Hepes-KOH at pH 7, pH 7.5 or pH 8, or 0.1 M Bicine-KOH at pH 8, pH 8.5 or pH 9, each with 1 mM EDTA.

The activity of PPH towards different substrates was determined by incubating 150 µg ml^-1^ PPH with 200 µM substrates or 100 µM for pheophytin *b*. Substrates were obtained as follows: pheophytin *b* and bacteriopheophytin *a* were generated from chlorophyll *b* (Sigma-Aldrich) and bacteriochlorophyll *a* (Livchem Logistics), respectively, as previously described for pheophytin *a* (Schelbert *et al.*, 2009); pheophorbide *a* methyl ester and Zn(II) pheophorbide *a* methyl ester were gifts from Bernhard Kräutler at the University Innsbruck, Austria; pyropheophorbide *a* methyl ester and protoporphyrin IX dimethyl ester were from Livchem Logistics. For calculating the K_M_ values for pheophytin *a*, pheophorbide *a* methyl ester, bacteriopheophytin *a*, and pheophytin *b* different substrate concentrations, as shown in the Figures, were incubated with 150 µg ml^-1^ PPH for 30 min. The K_M_ values were calculated with Prism (GraphPad).

For testing the mutated PPH variants, expressed proteins at a concentration of 150 µg ml^-1^ were incubated with 200 µM pheophytin *a* for 60 min.

### Activity of PPH on isolated thylakoid membranes

An Arabidopsis *pph-1*/*clh1-1*/*clh2-1* triple mutant was produced by crossing a *clh1-1*/*clh2-1* double mutant (Schenk *et al.*, 2007) with *pph-1* (Schelbert *et al.*, 2009) Plants were grown for 4 weeks under long-day conditions, namely 16 h light and 8 h dark, at 22°C during the day and 18°C at night, with a fluence rate of 100 to 200 µmol photons m^-2^ s^-1^ and 60% relative humidity. Thylakoid membranes were isolated from green or senescent leaves after 5 d of dark incubation according to published procedures (Oh *et al.*, 2003; Schelbert *et al.*, 2009). In brief, leaves were ground in a Sorvall mixer with grinding buffer, comprising 50 mM Hepes-KOH at pH 7.6, 0.3 M sorbitol, 10 mM NaCl, and 5 mM MgCl_2_; 20 ml of buffer was used per 0.9–1 g leaf material. Next this mixture was filtered through double-layered gauze. The homogenized tissue was centrifuged at 20 000 g for 5 min at 4°C and the supernatant discarded. The pellets were washed twice by gently resuspending in breakage buffer, comprising 50 mM Hepes-KOH at pH 7.6, 0.1 M sorbitol, 10 mM NaCl, and 5 mM MgCl_2_; 4 ml of buffer was used per 1 g leaf material. The mixture was then centrifuged. The pellets were washed twice more in reaction buffer, comprising 0.1 M Bicine-KOH at pH 8.5 and 1 mM EDTA. The concentration of isolated membranes was adjusted in reaction buffer to 250 µg chlorophyll *a* ml^-1^ for green leaves and to 180 µg chlorophyll *a* ml^-1^ for senescing leaves. For this, chlorophyll was quantified as previously published (Strain *et al.*, 1971). Finally, thylakoid membranes were 1:1 [v/v] mixed with reaction buffer without Triton X100 or with reaction buffer containing 1% [v/v] Triton X100 to reach a final Triton X100 concentration of 0.5%. Membranes were incubated at 4°C for 30 min and then directly used for activity assays. Activity assays were performed by incubating 75 µl membranes with 25 µl PPH (75 µg) at 25°C for 120 min. Reactions were stopped by adding three volumes [v/v] of acetone and then analyzed by HPLC.

### Modeling and structural analysis of ΔPPH and CLH1

Modeling of PPH without the predicted N-terminal transit peptide (ΔPPH) and CLH1 was performed with the Protein Homology/analogY Recognition Engine V 2.0 (Phyre2; http://www.sbg.bio.ic.ac.uk/phyre2/, last accessed 6 September 2017) (Kelley *et al.*, 2015). The following protein structures, denoted by RCSB PDB identifiers, were used by Phyre2 to model ΔPPH: 3I28, 1CR6, 4D9J, 4QLO, 2B61, 2VAV, 2QMQ, 3I1I, 2Y6V, 5D6O, 2VVL, 2VAT, 5F47, 2PL5, 4F0J, 4QLA, 4I19, 5EF7, 2E3J, 3OOS. The following protein structures, denoted by RCSB PDB identifiers, were used by Phyre2 to model CLH1: 3D59, 3VIS, 2FX5, 1JFR, 4EB0, 4WFI, 3HLK, 2ECF, 3K2I, 2B9V, 1JU3, 3AZQ, 2JBW.A, 1L7Q, 2JBW.B, 1MPX, 1L7A, 4ZRS, 2B9V, 3MVE. The obtained models were visualized using the Discovery Studio Visualizer software (Dassault Systems). Protein secondary structure prediction was done with the PSIPRED Protein Sequence Analysis Workbench (http://bioinf.cs.ucl.ac.uk/psipred/, last accessed 6 September 2017).

### Phylogenetic analysis

Arabidopsis PPH orthologs were identified with BLASTP searching (Altschul *et al.*, 1997) the Phytozome database (https://phytozome.jgi.doe.gov, last accessed 6 September 2017). Accession numbers are as follows: *Aquilegia coerulea*: Acoe 1, 22028438; Acoe 2, 22028440; Acoe 3, 22028441; *Arabidopsis lyrata*: Alyr, 16059924; *Arabidopsis thaliana*: Atha (AT5G13800), 19671250; *Amborella trichopoda*: Atri, 31572443; *Brachypodium distachon*: Bdis 1, 32791837; Bdis 2, 32791838; *Brassica rapa*: Brap 1, 30607511; Brap 2, 30617286; *Brachypodium stacei*: Bsta, 32872976; *Boechera stricta*: Bstr, 30656110; *Citrus clementina*: Ccle, 20803863; *Capsella grandiflora*: Cgra, 28914601; *Carica papaya*: Cpap, 16412351; *Capsella rubella*: Crub, 20908496; *Cucumis sativus*: Csat 1, 16962141; Csat 2, 16962144; *Citrus sinensis*: Csin 1, 18102612; Csin 2, 18102614; *Eucalyptus grandis*: Egra 1, 32071411; Egra 2, 32071412; *Eutrema salsugineum*: Esal, 20205993; *Fragaria vesca*: Fves, 27256941; *Glycine max*: Gmax 1, 30485515; Gmax 2, 30548341; Gmax 3, 30535348; *Gossypium raimondii*: Grai 1, 26771977; Grai 2, 26816354; *Kalanchoe marnieriana*: Kmar 1, 32566857; Kmar 2, 32566858; Kmar 3, 32576249; *Linum usitatissimum*: Lusi 1, 23154596; Lusi 2, 23139227; *Musa acuminata*: Macu, 32295673; *Malus domestica*: Mdom 1, 22644010; Mdom 2, 22677109; Mdom 3, 22645276; *Mimulus guttatus*: Mgut 1, 28947539; Mgut 2, 28924057; *Medicago truncatula*: Mtru, 31060299; *Oryza sativa*: Osat 1, 33147660; Osat 2, 33147661; Osat 3, 33147662; *Panicum hallii*: Phal, 32519143; *Physcomitrella patens*: Ppat 1, 32980877; Ppat 2, 32980880; Ppat 3, 32980879; *Prunus persica*: Pper, 32074354; *Populus trichocarpa*: Ptri 1, 26988021; Ptri 2, 26988022; *Panicum virgatum*: Pvir 1, 30302578; Pvir 2, 30302579; Pvir 3, 30222197; Pvir 4, 30222200; Pvir 5, 30222198; Pvir 6, 30222199; Pvir 7, 30222201; Pvir 8, 30264911; Pvir 9, 30264913; *Phaseolus vulgaris*: Pvul 1, 27148885; Pvul 2, 27150655; *Ricinus communis*: Rcom, 16824129; *Sorghum bicolor*: Sbic, 32744955; *Setaria italica*: Sita, 32702516; *Solanum lycopersicum*: Slyc, 27302437; *Spirodela polyrhiza*: Spol, 31513730; *Salix purpurea*: Spur 1, 31398042; Spur 2, 31439261; Spur 3, 31439264; *Solanum tuberosum*: Stub 1, 24421437; Stub 2, 24421436; *Setaria viridis*: Svir, 32661223; *Theobroma cacao*: Tcac, 27432758; *Vitis vinifera*: Vvin, 17817618; *Zea mays*: Zmay 1, 30984753; Zmay 2, 30984754. Additional sequences for PPH-like proteins of Arabidopsis (Lin *et al.*, 2016) were retrieved from TAIR (http://www.arabidopsis.org/, last accessed 6 September 2017): At4g36530; At5g19850; At5g38520.

Multiple sequence alignment of these proteins was generated and phylogenetic analysis (Supplementary Fig. S5B) performed with the neighbor-joining method using MEGA7 (Kumar *et al.*, 2016). Bootstrap analysis was performed with 1000 replicates. For Supplementary Fig. S5A, the multiple protein alignment excluding the three PPH-like proteins of Arabidopsis, which compared with all other sequences (> 52.6%) share much lower identity with Arabidopsis PPH (25.1–31.1%), was analyzed using WebLogo (http://weblogo.threeplusone.com/, last accessed 6 September 2017).

## Results

### PPH is a high-affinity pheophytinase

**Fig. 1.**
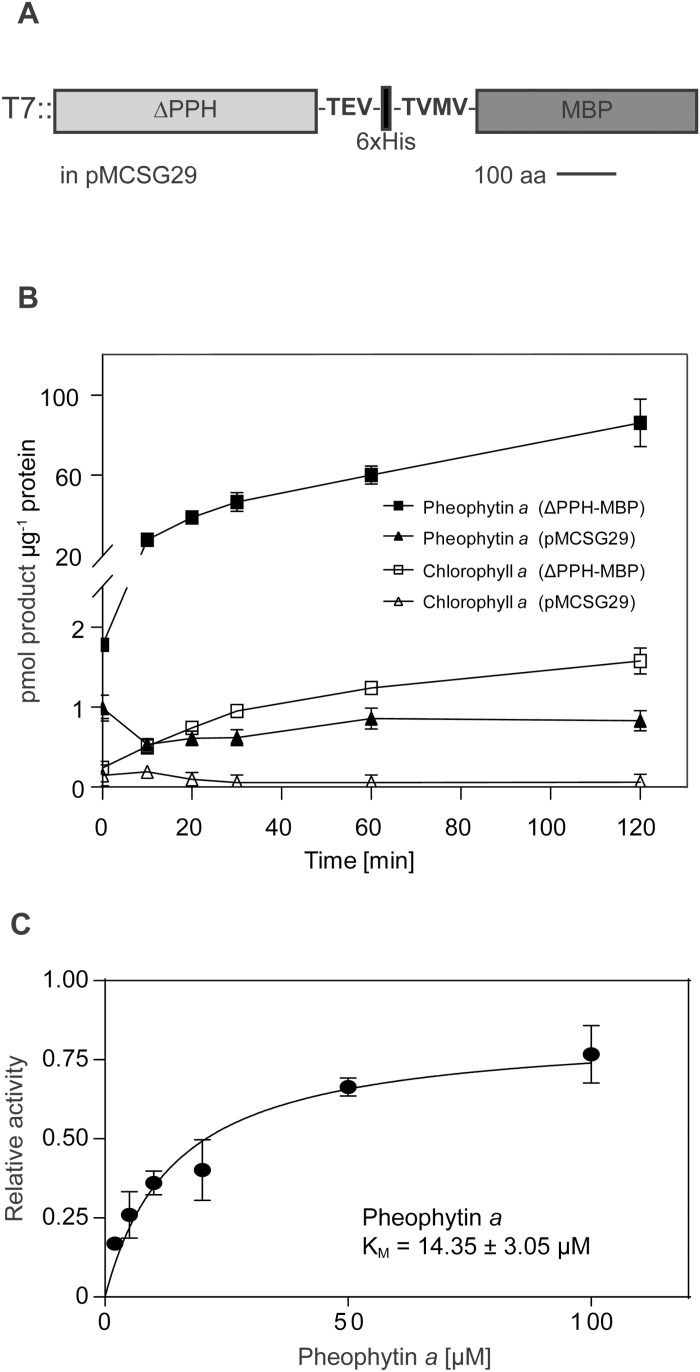
Biochemical properties of PPH. (A) Schematic overview of the ∆PPH-MBP fusion protein used in this work. (B) Time-dependent formation of pheophorbide *a* and chlorophyllide *a* from pheophytin *a* and chlorophyll *a*, respectively, in assays with ∆PPH-MBP or vector control (pMCSG29). Note that the activity of ∆PPH-MBP with chlorophyll *a* as substrate was very low. (C) Activity of ∆PPH-MBP on pheophytin *a* followed Michaelis-Menten kinetics with an apparent K_M_ of 14.35 µM. All data are mean values ± standard deviation of three replicates.

Arabidopsis PPH consists of 484 amino acids, including a predicted 46 amino acid long chloroplast transit peptide (Schelbert *et al.*, 2009). In order to determine its biochemical characteristics, a truncated form, lacking the transit peptide (∆PPH), was cloned into pMCSG29 (Eschenfeldt *et al.*, 2010) resulting in a ∆PPH-MBP fusion protein (Fig. 1A). The following biochemical investigations described were carried out with this protein form. After chemical induction of protein expression in *E. coli*, ∆PPH-MBP was present in the soluble protein fraction and was directly used for determining hydrolytic activity as activities of crude extracts and purified enzyme were indistinguishable. The optimal temperature of ∆PPH-MBP was found to be 34°C (Supplementary Fig. S1A) and the optimal pH optimum to be 8.5 (Supplementary Fig. S1B). As already published (Schelbert *et al.*, 2009; Guyer *et al.*, 2014), ∆PPH-MBP showed high hydrolytic activity towards pheophytin *a* (Fig. 1B), while activity against chlorophyll *a*, which structurally resembles pheophytin *a* but contains a central Mg atom, was very low (Fig. 1B). The activity of ∆PPH-MBP on pheophytin *a* followed Michaelis-Menten kinetics with an apparent K_M_ of 14.4 µM, indicating PPH performs hydrolysis of its natural substrate with high affinity (Fig. 1C). To investigate whether chlorophyll may compete with pheophytin for substrate binding to PPH, we determined the kinetics of pheophytin *a* hydrolysis in the presence of chlorophyll *a* (Supplementary Fig. S2). This analysis confirmed that chlorophyll is indeed able to inhibit ∆PPH-MBP activity at low pheophytin *a* concentrations, indicating competition for binding by PPH; but at saturating substrate concentrations, this inhibition was out competed. Due to saturation of substrate and inhibitor solubility, the type of inhibition could not be determined.

### PPH is not active on pheophytin embedded within thylakoid membranes

**Fig. 2.**
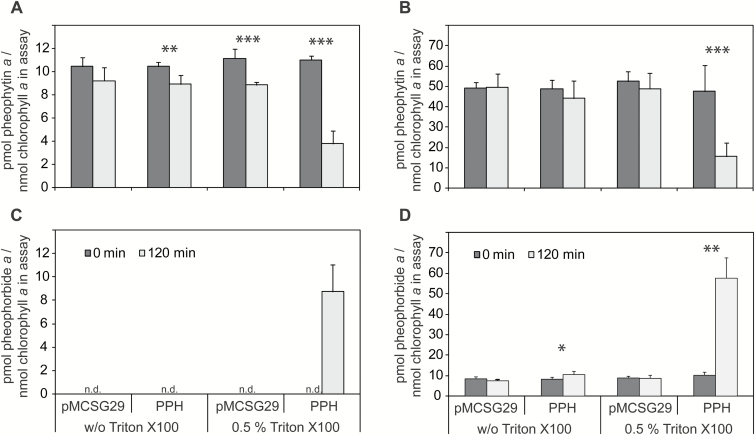
Phytol hydrolytic activity of PPH with pheophytin *a* embedded within thylakoid membranes. (A, B) Pheophytin *a* content relative to chlorophyll *a* content in thylakoid membranes isolated from leaves of the *pph-1*/*clh1-1*/*clh2-1* triple mutant before (A) and after 5 d of dark-induced senescence (B). (C, D) Formation of pheophorbide *a* in assays with recombinant ∆PPH-MBP using unsolubilized (w/o Triton X100) or partially solubilized (0.5% Triton X100) thylakoid membranes before (C) and after (D) senescence induction. Note that ∆PPH-MBP is only active when membranes were solubilized. All data are mean values ± standard deviation of three biological replicates, each with 1–2 technical replicates. Asterisks indicate significantly different values after 120 min compared with 0 min; Student’s *t* test; n.d., not detected. *P≤0.05; **P≤0.01; ***P≤0.001.

The activity tests with ∆PPH-MBP described above were carried out *in vitro* with soluble pheophytin *a* as a substrate. However *in vivo*, as the primary electron acceptor of P680 in photosystem II (Krieger-Liszkay, 2005), pheophytin *a* is embedded within protein complexes in the thylakoid membrane. Similarly during senescence, dephytylation of chlorophyll is assumed to take place on pigments that are still embedded in the membrane and only after phytol hydrolysis is pheophorbide *a* sufficiently hydrophilic to potentially leave the complexes. In order to obtain a better understanding of PPH activity under more natural conditions, the hydrolytic activity of recombinant ∆PPH-MBP was tested by offering isolated thylakoid membranes that contain pheophytin as substrates. To this end, thylakoid membranes were isolated from green leaves of a *pph-1*/*clh1-1*/*clh2-1* triple mutant. The triple mutant was chosen to abolish endogenous PPH activity and to prevent unspecific formation of chlorophyllide by either CLH1 and/or CLH2 (Schenk *et al.*, 2007). Isolated membranes were directly incubated with recombinant ∆PPH-MBP or were pre-treated with 0.5% Triton X-100 to partially solubilize them (Fig. 2). Surprisingly, ∆PPH-MBP was incapable of hydrolyzing pheophytin from non-solubilized membranes of green leaves (Fig. 2C). However, after partial membrane solubilization ∆PPH-MBP gained access to its substrate and pheophorbide formation could be recorded (Fig. 2C). Similar results were obtained when using thylakoid membranes isolated from senescing leaves. Thus, despite the fact that plastid membranes from senescent *pph-1*/*clh1-1*/*clh2-1* leaves contained higher quantities of pheophytin *a* (Fig. 2A, B), ∆PPH-MBP was exclusively active after membrane solubilization (Fig. 2D). These results may be explained by rather tight thylakoid membrane stacking in the plastids of PPH-deficient plants as shown earlier (Schelbert *et al.*, 2009), likely preventing PPH from gaining access to its substrate.

### PPH is an esterase with specificity for the acidic moiety of the ester bond

**Fig. 3.**
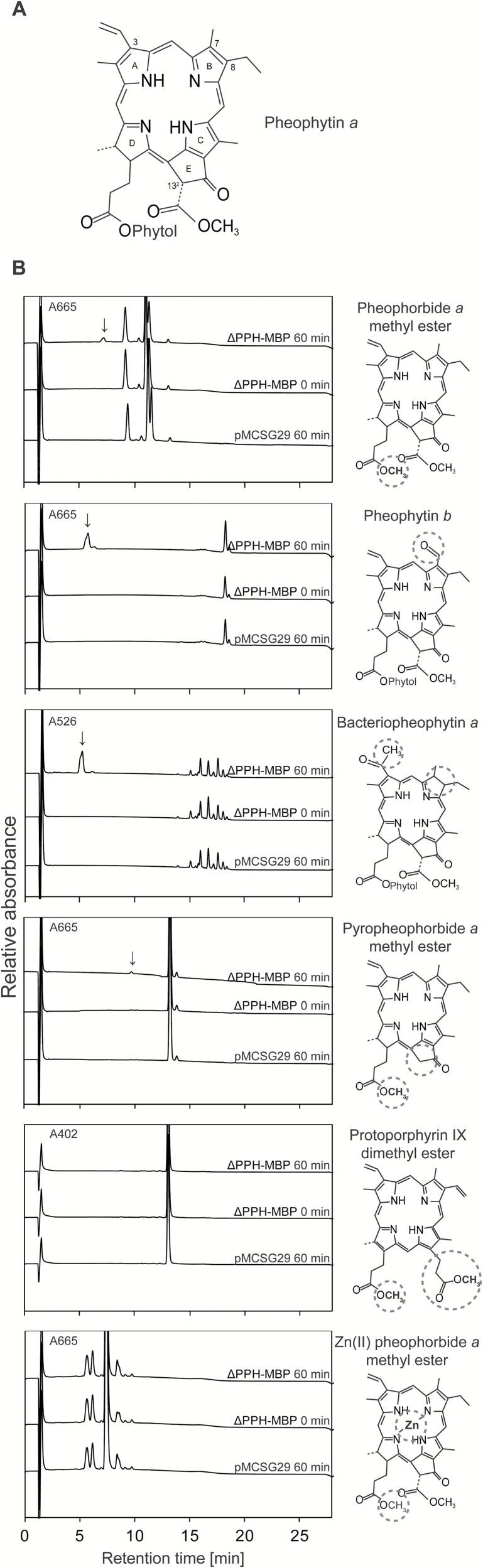
Activity of PPH on different porphyrin substrates. (A) Chemical structure of pheophytin *a*. (B) HPL chromatograms of activity assays with ∆PPH-MBP after 0 min and 60 min, and with the vector control (pMCSG29) after 60 min of incubation. Product formation is indicated with arrows. Substrate structures are shown on the right and structural differences as compared with pheophytin *a* are indicated with circles.

The high substrate specificity of ∆PPH-MBP for pheophytin *a* as compared with chlorophyll *a* (Fig. 1) is rather striking, considering that absence or presence of the central Mg is the only difference between these two pigments. To further explore which parts of the pigment determine the substrate specificity of PPH, different pheophytin derivates were tested as substrates. It is known that many esterases are specific for either the alcohol or the acid moiety of the ester bond (Fojan *et al.*, 2000). We therefore tested substrates that had modifications in the porphyrin ring, namely the acidic moiety, or differed in the esterified phytyl chain, namely the alcohol moiety (Fig. 3; see Supplementary Fig. S3 for absorption spectra of respective substrates and products).

**Fig. 4.**
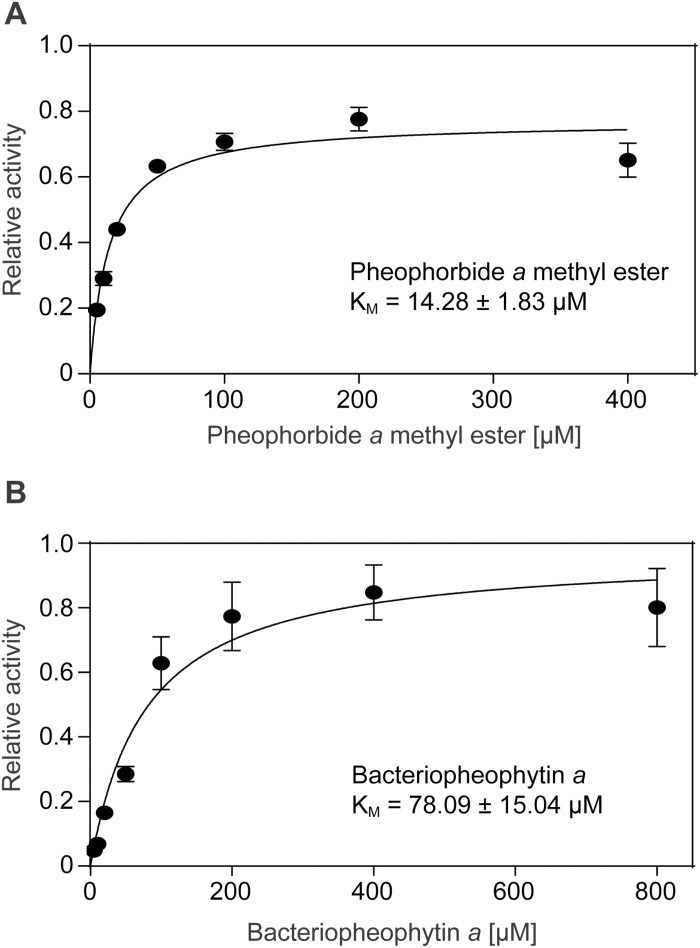
Determination of the K_M_ values for pheophorbide *a* methyl ester and bacteriopheophytin *a*. Activity of ∆PPH-MBP with pheophorbide *a* methyl ester (A) and bacteriopheophytin *a* (B) followed Michaelis-Menten kinetics. All data are mean values ± standard deviation of three replicates.

Preference of ∆PPH-MBP for the alcohol moiety was tested with pheophorbide *a* methyl ester, where the hydrophobic phytol chain is replaced by a methyl group. The results show that the length of the alcohol chain has no influence on activity and that the methyl group is hydrolyzed with an efficiency comparable to the phytyl group (Fig. 3B). This was corroborated by enzyme kinetic tests of ∆PPH-MBP with pheophorbide *a* methyl ester (Fig. 4A). As for pheophytin *a*, activity followed Michaelis Menten kinetics with a similar apparent K_M_ value of 14.3 µM, implying that the alcohol moiety of the substrate seems rather irrelevant for ∆PPH-MBP activity.

Next, we assessed the requirements of ∆PPH-MBP regarding the porphyrin moiety of the substrate. First, pheophytin *b* was tested. Since PAO, the downstream enzyme of PPH during chlorophyll breakdown, only accepts pheophorbide *a* (Hörtensteiner *et al.*, 1995), and because ‘*b*’ to ‘*a*’ conversion was shown to take place at the level of chlorophyll (Shimoda *et al.*, 2012), pheophytin *b* is likely not a natural substrate of PPH. Nevertheless *in vitro*, the C7-formyl group of pheophytin *b* does not abolish ∆PPH-MBP activity and pheophorbide *b* is formed (Fig. 3B). However, in contrast to pheophytin *a* (Fig. 1C), pheophorbide *b* did not reach saturation in the tested concentration range (Supplementary Fig. S4), which meant a K_M_ value could not be determined. Thus, affinity of PPH for pheophytin *b* seems significantly lower than that for pheophytin *a*. Similar results were found for bacteriopheophytin *a*, which, in comparison to pheophytin *a*, has a reduced C7/C8 double bond and an acetyl group instead of a vinyl group at C3 of the pyrrole ring A. ∆PPH-MBP was able to form bacteriopheophorbide *a* (Fig. 3B) but, with an apparent K_M_ value of 78.1 µM (Fig. 4B), the affinity for bacteriopheophytin *a* was about five times lower compared with pheophytin *a*. From these findings we conclude that the ‘northern’ part of the porphyrin ring structure is rather flexible, allowing for PPH activity, but with reduced affinity. By contrast, pyropheophorbide *a* methyl ester, where the C13^2^ carboxymethyl group of the pheophorbide *a* methyl ester is absent, was a very weak substrate for ∆PPH-MBP (Fig. 3B) and enzyme kinetics could not be determined. Similarly, protoporphyrin IX dimethyl ester, the esterified precursor of chlorophyll and heme biosynthesis that lacks the isocyclic ring E, was not accepted as a substrate (Fig. 3B). Assays using Zn(II) pheophorbide *a* methyl ester did not result in product formation (Fig. 3B). This confirmed the prerequisite of a metal-free porphyrin ring; in line with the fact that chlorophyll is only a very weak PPH substrate. From all these results it can be concluded that PPH is an esterase with specificity for the acidic moiety of the ester bond, while the nature of the alcohol is not relevant.

### PPH is an α/β hydrolase with a catalytic triad composed of serine, histidine, and aspartate

**Fig. 5.**
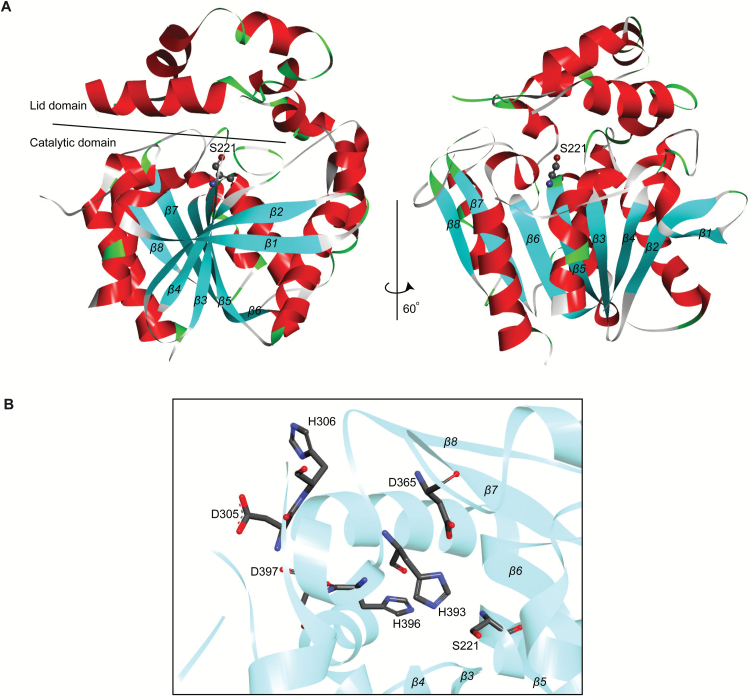
Three-dimensional model of ΔPPH. (A) Cartoon representation of the Phyre2 model of ΔPPH. *β* strands (*β*1–*β*8) and α helices are shown in blue and red, respectively. The catalytic serine residue is indicated. (B) Close-up view of the active site of ΔPPH, showing the catalytic triad residues (S221, D365 and H393 or H396) and further residues analyzed in this work by site-directed mutagenesis.

Arabidopsis PPH is known to be a α/β hydrolase with serine 221 (S221) in its active site (Schelbert *et al.*, 2009). In most α/β hydrolases, activity depends on two additional crucial residues, namely histidine and aspartate, which together with the active-site serine form the so-called catalytic triad (Rauwerdink and Kazlauskas, 2015). However, the histidine and aspartate residues forming the catalytic triad in PPH have not yet been identified. With the help of the web-based program Phyre2 (Kelley *et al.*, 2015) the 3-dimensional structure of ∆PPH, namely without the N-terminal chloroplast signal sequence, could be modeled based on comparison with several α/β hydrolases for which crystal structures have been determined in the past (see Material and Methods). The model confirms PPH as a classical α/β hydrolase fold enzyme with a catalytic domain that is composed of a central β sheet of eight strands, which are interconnected by α helices (Rauwerdink and Kazlauskas, 2015). As in other α/β hydrolases, PPH also possesses a lid domain with the active serine residue lying between the two domains at the surface of the catalytic domain (Fig. 5A). The lid domain of PPH exclusively consists of α helices and is inserted between strands β6 and β7, as in many other serine hydrolases (Rauwerdink and Kazlauskas, 2015).

**Fig. 6.**
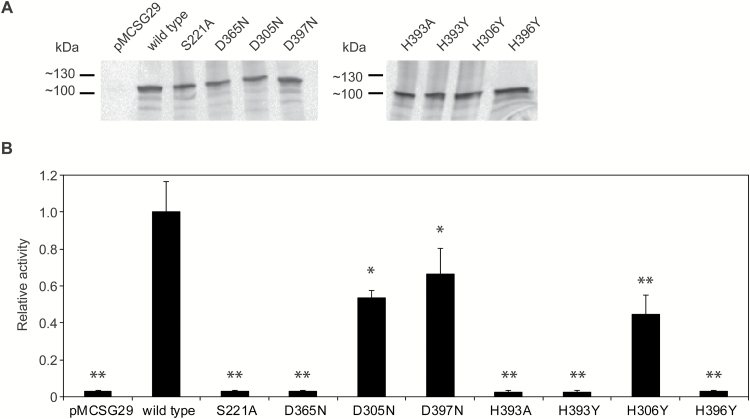
Activity of site-directed mutated PPH. (A) Immunoblot analysis of soluble fractions of recombinant PPH proteins probed with anti-PPH antibodies. (B) Activity analysis of recombinant PPH proteins with pheophytin *a*. Data are mean values ± standard deviation of three replicates. Asterisks indicate significantly lower activity as compared with the wild-type enzyme; Student’s *t* test; *P≤0.05; **P≤0.01. pMCSG29, vector control; wild-type, ∆PPH-MBP.

The phylogenetic tree in Supplementary Fig. S5B shows the wide distribution of highly homologous PPH proteins within land plants. Using the model (Fig. 5B) and a sequence alignment of PPH proteins from these different plant species (web-logo; Supplementary Fig. S5), we predicted the conserved residues aspartate 365 (D365) and histidine 393 (H393) or H396 of Arabidopsis PPH to be part of the catalytic triad (Fig. 5). In order to test this, PPH proteins that are mutated in these residues were generated by site-directed mutagenesis and analyzed for their activity (Fig. 6). The polar S221 was mutated to a nonpolar alanine (S221A), as already published (Schelbert *et al.*, 2009) and the charged D363 to an uncharged asparagine (D363N). For H393 two mutants were generated, namely to a polar alanine (H393A) or to a nonpolar tyrosine (H393Y); likewise H396 was changed to tyrosine (H396Y). As controls, aspartate 305 (D305), D397, and histidine 306 (H306) that locate close to the active site but on the lid domain (Fig. 5B), were mutated to asparagine (D305N; D397N) and tyrosine (H306Y), respectively. All mutated sequences were expressed in pMCSG29 (Eschenfeldt *et al.*, 2010) as N-terminally truncated MBP fusions as described above for wild-type PPH (∆PPH-MBP) (Fig. 6A and Supplementary Fig. S7). Activities were tested with pheophytin *a* and pheophorbide *a* formation was analyzed by HPLC (Fig. 6B). All proteins with mutations in a residue of the predicted catalytic triad had entirely lost their activity, while the control mutants, D305N, D397N and H306Y, retained around 60% of wild-type PPH activity (Fig. 6B). This confirmed that these latter two residues are not part of the catalytic triad of PPH, which is composed of S221, D365, and H393 or H396.

## Discussion

Metabolism of chlorophyll is among the few biochemical processes in plants that can be visually perceived by laymen and scientists alike, and perception of chlorophyll degradation during autumn in deciduous trees or during fruit ripening may be as old as mankind. It is not overly surprising, therefore, that the first ever described plant enzyme is CLH, which hydrolyses the phytol moiety of chlorophyll. Since its first discovery in 1913 (Willstätter and Stoll, 1913), CLH was considered to be involved in the pathway of chlorophyll degradation (Takamiya *et al.*, 2000); however, recently the *in vivo* requirement of CLH for chlorophyll breakdown was refuted or at least questioned (Schenk *et al.*, 2007; Hu *et al.*, 2015). Instead a novel hydrolase, PPH, was detected (Morita *et al.*, 2009; Schelbert *et al.*, 2009) and shown to specifically catalyze dephytylation of pheophytin, but not of chlorophyll, during leaf senescence. This specificity is remarkable but reasonable from a physiological point of view. Employment of PPH instead of CLH metabolically separates chlorophyll biosynthesis from degradation, thus avoiding chlorophyllide, the penultimate pigment in biosynthesis, becoming an intermediate of breakdown as well (Schelbert *et al.*, 2009). The present study attempted to elucidate the molecular and biochemical features of PPH in more detail.

Despite intensive attempts to purify and crystallize recombinant PPH, we were unsuccessful. Instead, we modelled the 3D structure of PPH based on publicly available structures of other α/β fold hydrolases. This model (Fig. 5) allowed the identification of residues in Arabidopsis PPH that constitute the catalytic triad, namely S221, D365, and H393. Using site-directed mutagenesis, these residues were confirmed to be critical for *in vitro* PPH activity. A S221A point mutation was previously shown to be unable to complement *pph-1* (Schelbert *et al.*, 2009). Besides H393, mutation of H396 also resulted in complete loss of activity (Fig. 6B). Thus, although H393 locates more closely between D365 and S221 than H396 (Fig. 5), we cannot exclude the possibility that H396 instead of H393 is part of the catalytic triad. All four residues are evolutionary conserved as deduced from the fact that they are present in all protein sequences of land plants used for phylogenetic analysis (Supplementary Fig. S5). As shown earlier (Schelbert *et al.*, 2009; Lin *et al.*, 2016), PPHs are likely also present in green algae, but not in cyanobacteria, and, thus, likely evolved after endosymbiosis.

Like other hydrolases, the catalytic mechanism of PPH likely requires, besides the catalytic triad, an oxyanion hole to stabilize a tetrahedral intermediate that forms during the hydrolase reaction (Rauwerdink and Kazlauskas, 2015). It is composed of at least two nitrogens of the amino acid backbone that provide hydrogen bonds to the oxyanion intermediate. Usually one of them is provided by the catalytic serine itself or from the residue next to it, while the other one is predicted to locate in the so-called oxyanion loop, between sheet β3 and the following α helix (Rauwerdink and Kazlauskas, 2015). Accordingly, we propose that one of the highly conserved residues between P123 and G128 (Supplementary Fig. S5A), most likely G124 (Supplementary Fig. S6A) that lies between β3 and the next α helix and is close to the catalytic serine, and L222 positioned next to it constitutes the oxyanion hole of PPH (Supplementary Fig. S6B).

The 3D model shows that PPH is composed of a catalytic domain with a classical α/β fold (Rauwerdink and Kazlauskas, 2015) and a rather extended lid domain. Interestingly, in a structural model of Arabidopsis CLH1 (Supplementary Fig. S8) this lid domain is almost absent, while the catalytic domain including the location of the catalytic triad with S138, D168, and H243 in CLH1 (Tsuchiya *et al.*, 2003) is rather similar to that of PPH, despite the low protein sequence identity of less than 25% between PPH and CLH1. It has been shown for several hydrolases that the lid domain adds to substrate binding and contributes to substrate specificity (Rauwerdink and Kazlauskas, 2015). Thus, it is very likely that the lid domain of PPH restricts its activity to certain metal-free porphyrins, in particular pheophytin, while in CLHs absence of a lid may set the basis for a rather wide substrate range. Indeed, recombinant *Citrus sinensis* CLH accepts both chlorophyll and pheophytin as substrates (Schelbert *et al.*, 2009). Likewise, CLHs from different plant species have been shown to efficiently hydrolyze a wide range of porphyrin esters, such as chlorophyll, pheophytin, pheophorbide methyl ester, Zn pheophytin, 13^1^-hydroxy pheophorbide methyl ester, and Zn pyropheophytin (McFeeters, 1975; Schoch and Ihl, 1998; Lee *et al.*, 2010), but also non-porphyrinic esters such as *p*-nitrophenyl decanoate (Arkus *et al.*, 2005). In addition, CLHs act as transesterases (Fiedor *et al.*, 1996), an activity that is absent in PPH. Again, this may to a large extent be due to the differences in the lid domain structure. It has to be noted that the recently identified chlorophyll dephytylase 1 (CLD1), which shares 27% sequence identity with PPH, has both chlorophyll- and pheophytin-hydrolyzing activity (Lin *et al.*, 2016). From its primary amino acid sequence (Lin *et al.*, 2016) and predicted secondary structure, CLD1 possesses an extended lid domain similar to that of PPH. Thus, substrate limitation in PPH may not exclusively be determined by the mere presence of the lid but by specific lid features that abolish activity of PPH towards metal-containing porphyrins. It will be interesting in the future to experimentally test whether insertion of the lid domain of PPH that is located between strands β6 and β7 (Supplementary Fig. S8C) may switch CLH1 to a pheophytinase or whether removing the lid domain in PPH or exchanging it with the lid domain of CLD1 would convert PPH to a hydrolase that accepts metal-containing porphyrins as substrate. Since the available structures of these enzymes are only based on models, this approach might however be challenging.

The lack of a crystal structure for PPH abolished the possibility to model the natural substrate of PPH, pheophytin *a*, into it, in order to potentially gain insights about which parts of the substrate are recognized by the enzyme. Instead, we used a biochemical approach to narrow down the structural requirements of porphyrinic compounds to serve as substrates for PPH. This analysis clearly demonstrated that the enzyme exclusively recognizes the porphyrin ring, chemically the acid moiety of the substrate, while the alcohol moiety seems irrelevant. Thus, the phytyl ester of pheophorbide *a*, namely the natural substrate pheophytin *a*, and the respective methyl ester were accepted by PPH with identical affinities (Figs. 1 and 4). From a physiological perspective, the lack of consideration of PPH towards the identity of the alcohol moiety is interesting because besides degradation of genuine chlorophyll it likely also allows hydrolysis of chlorophyll species that are conjugated with geranylgeraniol instead of phytol. Geranylgeranylated chlorophyll has been shown to be present in small quantities in wild-type Arabidopsis plants but to be massively increased in mutants that are deficient in light-harvesting complex like protein 3 (Tanaka *et al.*, 2010). By contrast, changing the acid part of the substrate significantly reduced or totally abolished PPH activity. In summary, our analysis demonstrated that the ‘southern’ part of the porphyrinic ring is most crucial for activity and activity was entirely lost in the absence of the isocyclic ring E (protoporphyrin IX dimethyl ester), while absence of the C13^2^ carboxymethylester (pyropheophorbide *a* methyl ester) largely reduced activity. This indicates that this ‘southern’ region, which is rather close to the actual site of hydrolysis, is highly important for substrate recognition by the enzyme. Changes to the ‘northern’ part of the porphyrin ring, as in bacteriopheophytin *a* and pheophytin *b*, also reduced the activity of PPH, but to less extent, indicating that recognition of this part of the substrate is less specific.

Interestingly, several of the substrates towards which PPH showed activity, namely pheophytin and pheophorbide methyl ester, have also been shown to be substrates for CLHs; nevertheless, the remarkable absence of PPH activity towards chlorophyll remains unexplained. Competition experiments demonstrated that, at high excess, chlorophyll can block the pheophytin *a* hydrolysis activity of PPH (Supplementary Fig. S2), indicating competition for substrate binding, which can be overcome by increasing pheophytin *a* concentrations. Unfortunately, we were unable to determine the type of inhibition, but we propose that the two substrates compete for binding at the active site in a competitive manner. If this is true, we must assume that presence of Mg in chlorophyll *a* abolished the catalytic cycle. A reason for this could lie in the inability of the enzyme to properly close the lid domain, when chlorophyll *a* is bound, to fully engage the substrate in the active site as a prerequisite for catalytic activity (Gregg *et al.*, 2015). Such steric hindrance could result from possible coordination of the Mg present in chlorophyll with certain amino acid residues within PPH. Indeed, for example all 35 chlorophyll molecules in the photosystem II monomer are coordinated through the Mg atom, seven of them by water, one by an asparagine residue, the rest by histidines (Umena *et al.*, 2011).

An additional interesting aspect of PPH relates to the spatial organization of the first steps of chlorophyll *a* breakdown that are catalyzed by two enzymes, which, based on their primary structure, are soluble proteins, namely SGR, the Mg dechelatase, and PPH, the phytol hydrolase (Hörtensteiner, 2009; Schelbert *et al.*, 2009; Shimoda *et al.*, 2016). To address this question we used recombinant PPH and investigated its ability to hydrolyze thylakoid membrane-bound pheophytin *a* (Fig. 2). Surprisingly, membrane-bound pheophytin *a* was totally inert towards recombinant PPH and only after partial membrane solubilization could the enzyme exert its activity. This is in contrast to non-yellow coloring 1-like, which catalyzes the first step of chlorophyll *b* to chlorophyll *a* reduction and which was shown to be able to extract pigments from isolated LHCII trimers during catalysis (Horie *et al.*, 2009). Our results indicate that *in vivo* activity of PPH might require the presence of further chlorophyll catabolic components, such as SGR and/or downstream chlorophyll catabolic enzymes (CCEs), or a certain spatial orientation of the enzymatic machinery and respective substrates, conditions that are not met in the performed assays. It is interesting to mention in this respect that CCEs have been proposed to physically interact during catalysis and together with LHCII subunits to constitute a highly dynamic complex that allows metabolic channeling of chlorophyll degradation intermediates (Sakuraba *et al.*, 2012). Also worth mentioning is the fact that, although showing a stay-green phenotype, *pph* mutants are not entirely blocked in chlorophyll breakdown and, accordingly, accumulate a certain amount of phyllobilins (Schelbert *et al.*, 2009). This indicates the presence of other hydrolases that function in addition to PPH in chlorophyll breakdown such as, for example, CLD1. Although *cld1* mutants do not exhibit a stay-green phenotype (Lin *et al.*, 2016), the activity of CLD1 or additional hydrolases could contribute to chlorophyll breakdown. This could also explain the presence of small amounts of pheophorbide in senescent thylakoid membranes of a *pph-1*/*clh1-1*/*clh2-1* triple mutant (Fig. 2D). However, biochemically CLD1 seems to be more similar to CLHs, as it is also capable of hydrolyzing both chlorophyll and pheophytin.

In summary, our work determined important biochemical and structural properties of PPH, a key enzyme in chlorophyll breakdown during leaf senescence. Modeling of its 3-dimensional structure combined with site-directed mutagenesis allowed the identification of the most important amino acid residues that compose the catalytic triad of the enzyme. Comparison of PPH with CLH identified important structural differences between these two classes of hydrolases that might explain their distinct catalytic properties.

## Supplementary Data

Supplementary data are available at *JXB* online.

Table S1. Primers used in this work.

Fig. S1. Temperature and pH optima of ∆PPH-MBP

Fig. S2. Partial inhibition of ∆PPH-MBP activity on pheophytin *a* by chlorophyll *a*.

Fig. S3. Absorbance spectra of substrates and their hydrolyzed products.

Fig. S4. Determination of the ∆PPH-MBP kinetics for pheophytin *b*.

Fig. S5. Amino acid distribution and phylogenetic analysis of PPH proteins in plants.

Fig. S6. The catalytic mechanism of PPH.

Fig. S7. Analysis of *E. coli* extracts expressing recombinant PPH proteins as described in Fig. 6.

Fig. S8. Three-dimensional model of CLH1 and comparison of ΔPPH and CLH1 secondary structures.

## Supplementary Material

supplementary_Table_S1_Figures_S1_S8

## Abbreviations:

CLD1: chlorophyll dephytylase 1
CLHs: chlorophyllases
PAO: pheophorbide *a* oxygenase
SGRs: stay-green proteins
