# Supplementary material for: Catalytic and structural properties of pheophytinase, the phytol esterase involved in chlorophyll breakdown
Source: J Exp Bot. 2017 Sep 23;69(4):879–89. doi: 10.1093/jxb/erx326 (PMC5853334; doi:10.1093/jxb/erx326)
Supplement: supplementary_Table_S1_Figures_S1_S8 [file erx326_suppl_supplementary_table_s1_figures_s1_s8.pdf]

Supplemental Table S1. Primers used in this work

Gene/construct	*Primer name*	*Sequence (5'-3')*
	Cloning of ΔPPH-MBP (pMCSG29)	
	PPH_LIC_fw	GTCTCTCCCATGAGTGGAAATTCCGATGGTTATG
	PPH_LIC_rv	GGTTCTCCCCAGCTGCAGACTTCCCTCCAAACAC
	Site-directed mutagenesis[1]	
PPH_D365N (w/o TP)	AtPPH_D365N_fw	GGAAGAGAAAATCCATGGGTG
	AtPPH_D365N_rv	CACCCATGGATTTTCTCTTCC
PPH_H393A (w/o TP)	AtPPH_H393A_fw	CCAGCGGGTGCCTGCCCACAC
	AtPPH_H393A_rv	GTGTGGGCAGGCACCCGCTGG
PPH_H393Y (w/o TP)	AtPPH_H393Y_fw	CCAGCGGGTTACTGCCCACAC
	AtPPH_H393Y_rv	GTGTGGGCAGTAACCCGCTGG
PPH_D305N (w/o TP)	AtPPH_D305N_fw	GTCTACACAAACCATTCTATC
	AtPPH_D305N_rv	GATAGAATGGTTTGTGTAGAC
PPH_H306Y (w/o TP)	AtPPH_H306Y_fw	GTCTACACAGACTATTCTATC
	AtPPH_H306Y_rv	GATAGAATAGTCTGTGTAGAC
PPH_H396Y (w/o TP)	AtPPH_H396Y_fw	CTGCCCATACGATGAAGTCCC
	AtPPH_H396Y_rv	GGGACTTCATCGTATGGGCAG
PPH_D397N (w/o TP)	AtPPH_D397N_fw	CTGCCCACACAATGAAGTCCC
	AtPPH_D397N_rv	GGGACTTCATTGTGTGGGCAG

[1]codons that define the point mutations are underlined

A

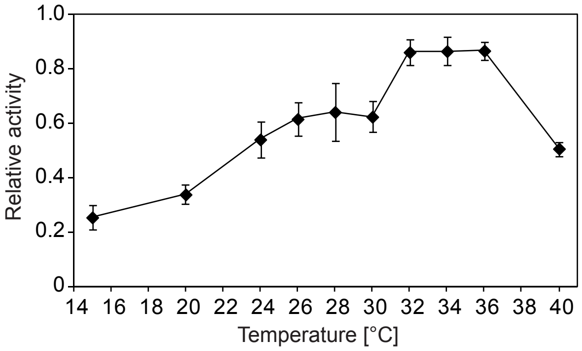

B

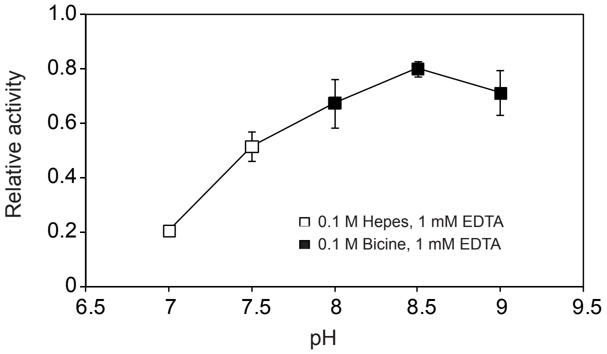

Supplemental Fig. S1. Temperature and pH optima of ΔPPH-MBP. ΔPPH-MBP activity with pheophytin *a* as substrate showed a temperature optimum of 34°C (A) and a pH optimum of pH 8.5 (B).

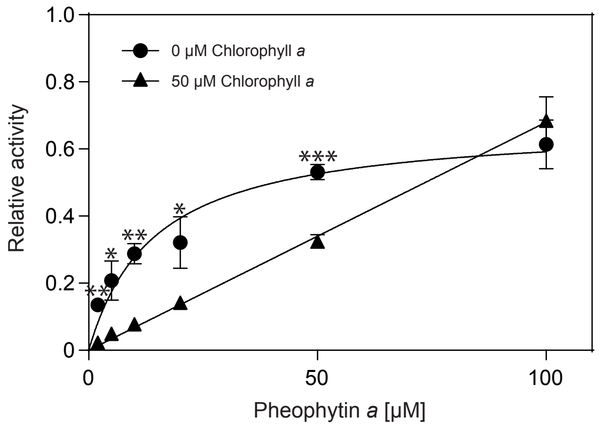

Supplemental Fig. S2. Partial inhibition of ΔPPH-MBP activity on pheophytin *a* by chlorophyll *a*. Assays were performed with increasing concentrations of pheophytin *a* in the absence (0 μM) or presence of 50 μM chlorophyll *a*. Note that it was not possible to determine the type of inhibition by chlorophyll *a* due to saturation of substrate solubility at higher concentrations. Asterisks indicate significantly lower values in the presence of chlorophyll compared to the absense of chlorophyll (Student's *t* test; $^{*}P \leq 0.05$; $^{**}P \leq 0.01$; $^{***}P \leq 0.001$)

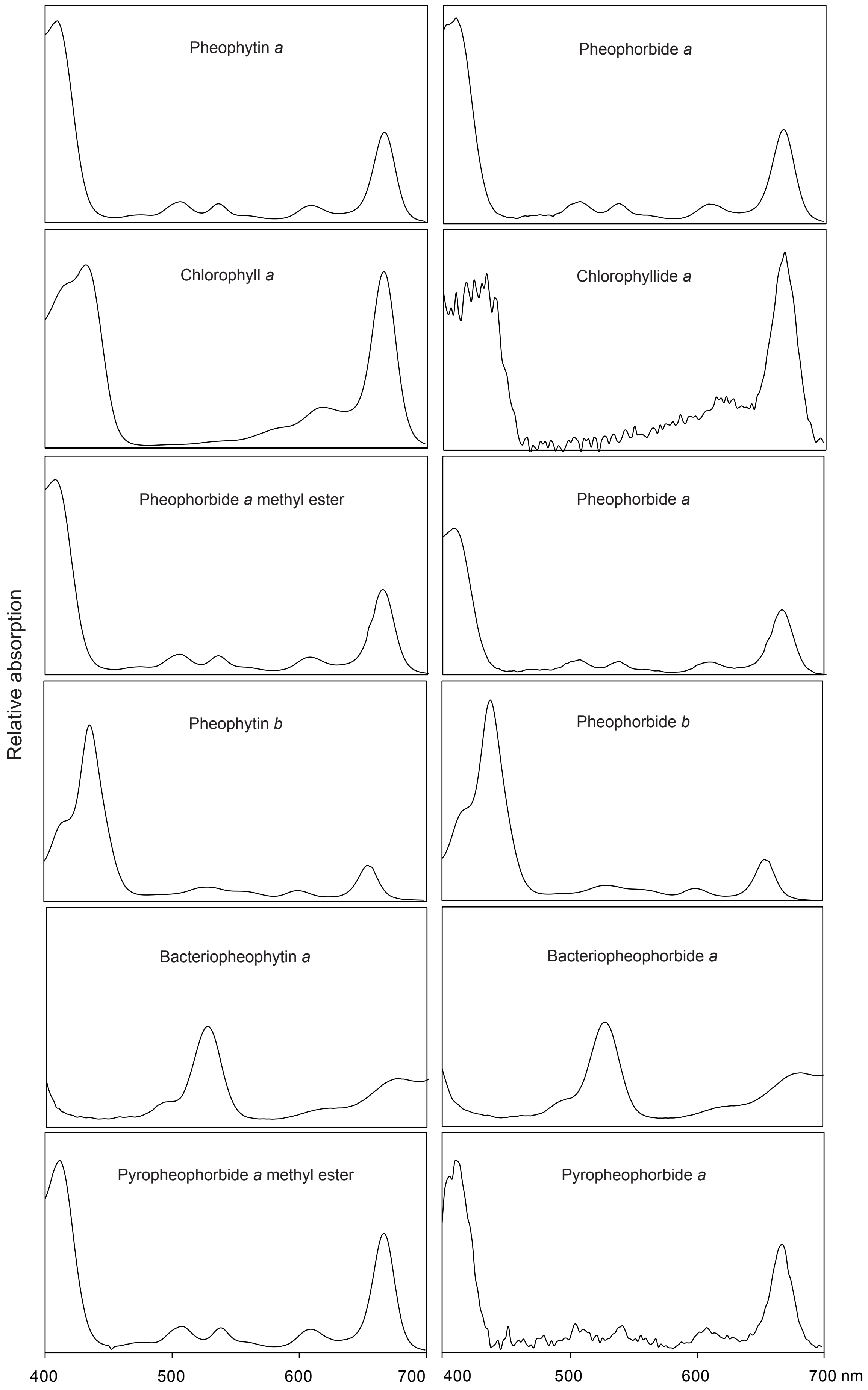

Supplemental Fig. S3. Absorbance spectra of substrates and their hydrolyzed products. HPLC spectra of substrates and products obtained in the assays performed in Fig. 3B are shown.

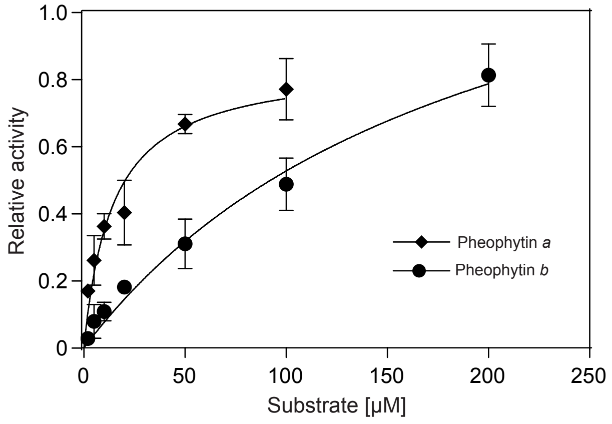

Supplemental Fig. S4. Determination of the ΔPPH-MBP kinetics for pheophytin *b*. Assays were performed with increasing concentrations of pheophytin *b*. Note that the data shown for pheophytin *a* are identical to Fig. 1C. Note also that calculation of a K_M for pheophytin *b* was not possible because activity was not saturated before reaching substrate solubility limits.

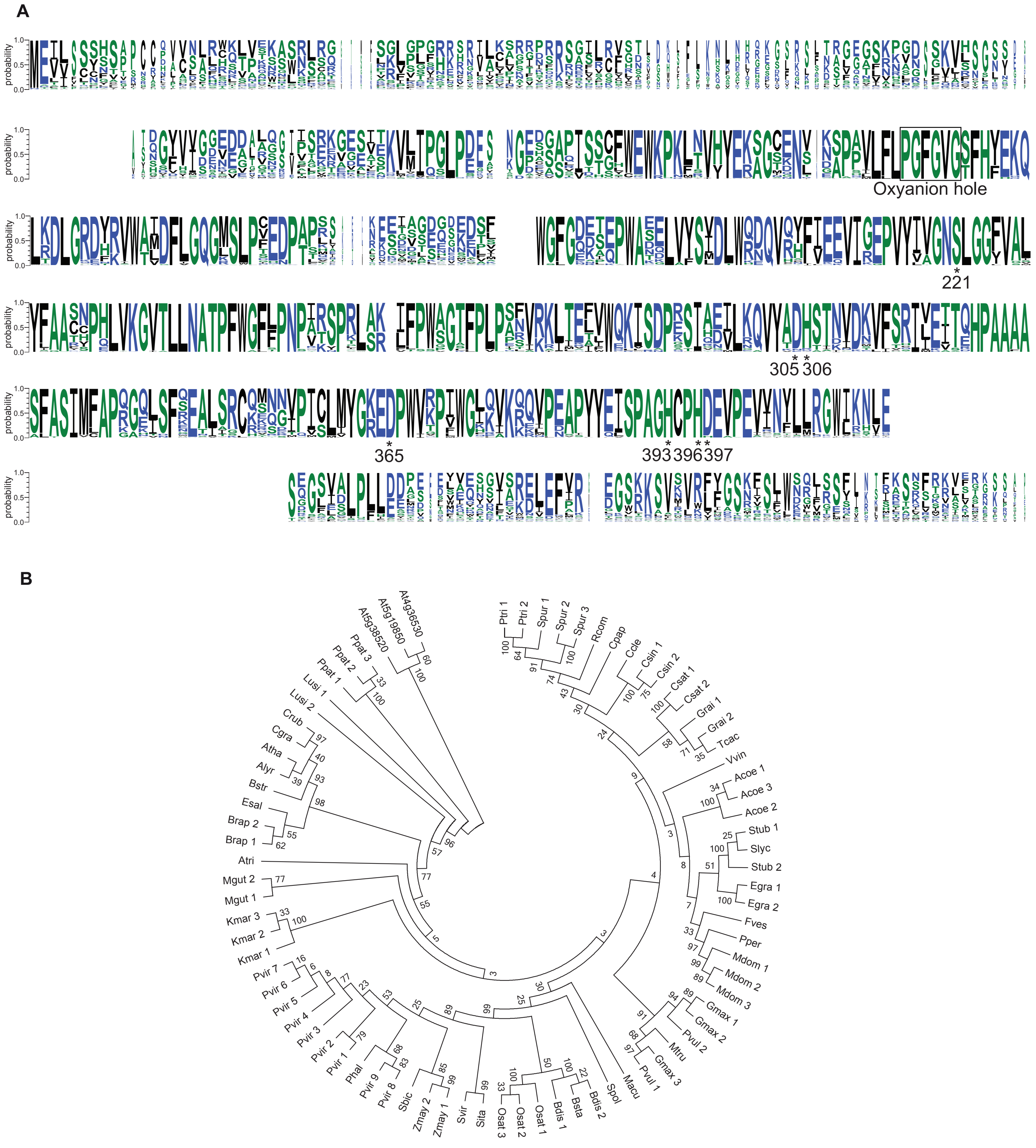

Supplemental Fig. S5. Amino acid distribution and phylogenetic analysis of PPH proteins in plants. (A) Graphical display of the amino acid distribution of a multiple sequence alignment of 77 PPH proteins from 43 plant species. The graphical output was produced using WebLogo (http://weblogo.threeplusone.com/). The amino acid residues mutated in this work and the likely region of the oxyanion hole of Arabidopsis PPH are labeled. (B) Phylogenetic analysis of PPH homologs in higher plants and of three PPH-like proteins of Arabidopsis (Lin et al., 2016). The tree was generated with the neighbor-joining method using MEGA7 (Kumar et al., 2016). Nodes are labeled with bootstrap values (% of 1000 replicates). Acor, *Aquilegia coerulea*; Alyr, *Arabidopsis lyrata*; Atha, *Arabidopsis thaliana*; Atri, *Amborella trichopoda*; Bdis, *Brachypodium distachon*; Brap, *Brassica rapa*; Bsta, *Brachypodium stacei*; Bstr, *Boechera stricta*; Ccle, *Citrus clementina*; Cgra, *Capsella grandiflora*; Cpap, *Carica papaya*; Crub, *Capsella rubella*; Csat, *Cucumis sativus;* Csin, *Citrus sinensis*; Egra, *Eucalyptus grandis*; Esal, *Eutrema salsugineum*; Fves, *Fragaria vesca*; Gmax, *Glycine max*; Grai, *Gossypium raimondii*; Kmar, *Kalanchoe marnieriana*; Lusi, *Linum usitatissimum*; Macu, *Musa acuminata*; Mdom, *Malus domstica*; Mgut, *Mimulus guttatus*; Mtru, *Medicago truncatula*; Osat, *Oryza sativa*; Phal, *Panicum hallii*; Ppat, *Physcomitrella patens*; Pper, *Prunus persica*; Ptri, *Populus trichocarpa*; Pvir, *Panicum virgatum*; Pvul, *Phaseolus vulgaris*; Rcom, *Ricinus communis*; Sbic, *Sorghum bicolor*; Sita, *Setaria italica*; Slyc, *Solanum lycopersicum*; Spol, *Spirodela polyrhiza*; Spur, *Salix purpurea*; Stub, *Solanum tuberosum*; Svir, *Setaria viridis*; Tcac, *Theobroma cacao*; Vvin, *Vitis vinifera*; Zmay, *Zea mays*.

A

B

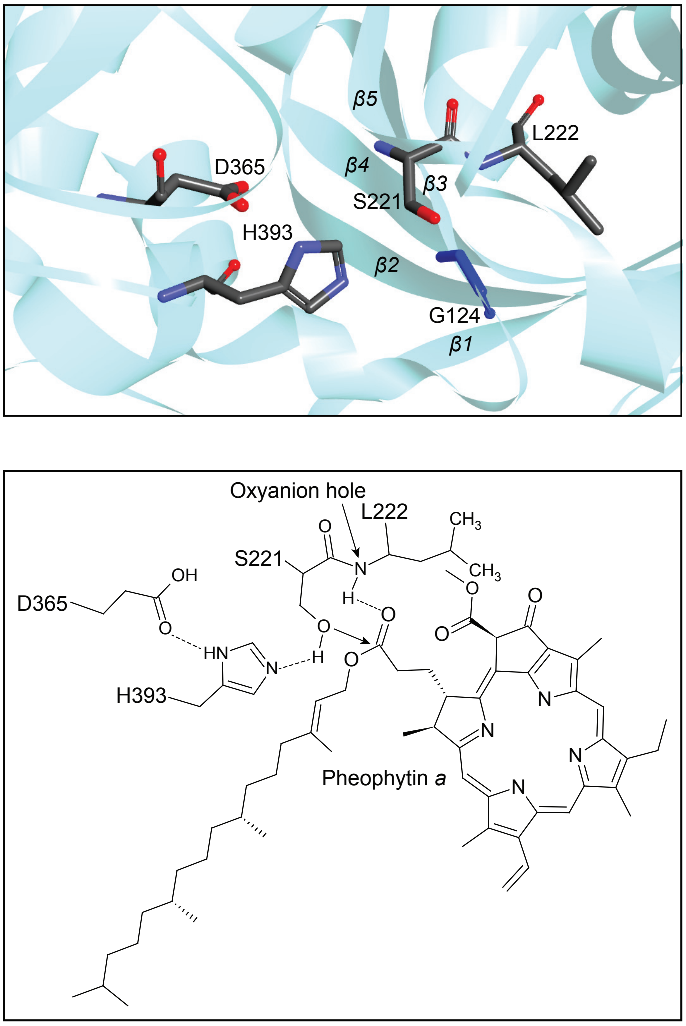

Supplemental Fig. S6. The catalytic mechanism of PPH. (A) Close-up view of the active site of the Phyre2 model of ΔPPH, showing the catalytic triad residues (S221, D365 and H393) and the likely oxyanion hole residues L222 and G124. Visible *β* strands are labeled. (B) Illustration of the nucleophilic attack of S221 on the ester bond of pheophytin *a*. Hydrogen bonds within the two other catalytic triad residues H393 and D365 and of the likely oxyanion hole residue L222 are indicated. Note that H396 that potentially could substitute H393 as a residue of the catalytic triad and the likely second oxyanion hole residue G124 are omitted.

A

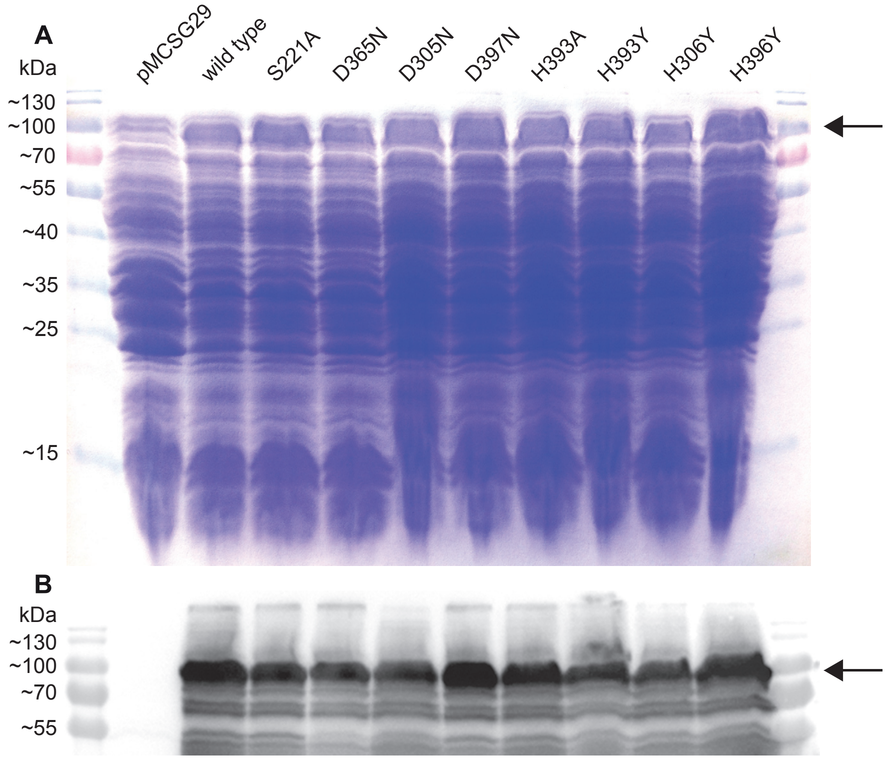

Supplemental Fig. S7. Analysis of *E. coli* extracts expressing recombinant PPH proteins as described in Fig. 6. (A) Commassie blue-stained SDS-PAGE gel. (B) Immunoblot analysis using anti-MPB antibodies. The arrows point to the ΔPPH-MBP fusions at a size of around 100 kDa.

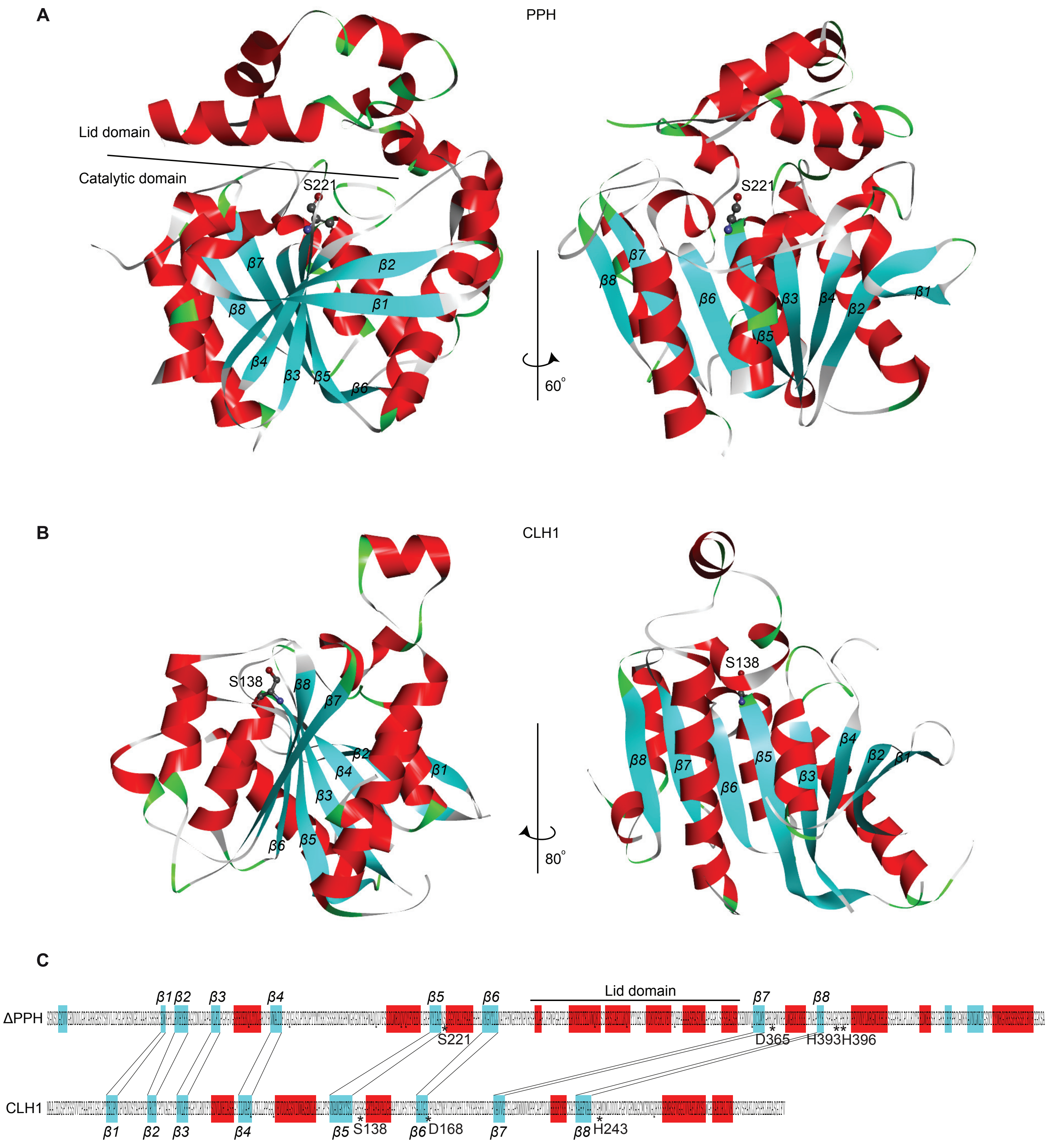

Supplemental Fig. S8. Three-dimensional model of CLH1 and comparison of ΔPPH and CLH1 secondary structures. (A) Cartoon representation of the Phyre2 model of ΔPPH (identical to Fig. 5A). (B) Cartoon representation of the Phyre2 model of CLH1. The catalytic serine residue (S138) is indicated. (C) Comparison of the secondary structures of ΔPPH and CLH1. Corresponding *β* strands are connected with lines. The residues of the catalytic triads of both proteins are labeled. For CLH1, these residues are according to published data (Tsuchiya et al., 2003). Note that the extended lid domain of ΔPPH is absent in CLH1. *β* strands (*β*1-*β*8) and α helices are shown in blue and red, respectively.
